# Three-dimensional evaluation of mandibular canine impaction characteristics and their relationship with lower incisor root morphometry

**DOI:** 10.4317/jced.59808

**Published:** 2022-10-01

**Authors:** Mabel Mejía-Milian, Luis-Ernesto Arriola-Guillén, Aron Aliaga-Del Castillo, Yalil-Augusto Rodríguez-Cárdenas, Gustavo-Armando Ruíz-Mora

**Affiliations:** 1 Division of Oral and Maxillofacial Radiology, School of Dentistry, Universidad Científica del Sur, Lima, Perú; 2 Division of Orthodontics and Division of Oral and Maxillofacial Radiology, School of Dentistry, Universidad Científica del Sur, Lima, Perú; 3 Postdoctoral Fellow. Department of Orthodontics, Bauru Dental School. University of São Paulo, Brazil; 4 Division of Oral and Maxillofacial Radiology, School of Dentistry, Universidad Científica del Sur, Lima, Perú. Associate Professor of the Division of Oral and Maxillofacial Radiology, School of Dentistry, Universidad Nacional de Colombia, Bogotá D.C, Colombia; 5 Division of Orthodontics, Faculty of Dentistry, Universidad Nacional de Colombia, Bogotá D.C, Colombia; and Associate Professor of the Division of Oral and Maxillofacial Radiology, School of Dentistry, Universidad Científica del Sur, Lima, Perú

## Abstract

**Background:**

To three-dimensionally evaluate the characteristics of mandibular canine impaction with the morphometry of the roots of the lower incisors.

**Material and Methods:**

Cone-beam computed tomography (CBCT) scans of 35 individuals (18 males and 17 females) with a mean age of 14.37± 10.26 years were evaluated. Forty-three impacted mandibular canines (IMbC) were evaluated. Their location, sector and level of impaction were determined. Likewise, the distances of the canines to the occlusal plane, the angulations with respect to the midline and adjacent lateral incisors, the length of the impacted canines, and of the lower incisors were measured. Finally, the presence of incisor root resorption was determined. Chi-square and Kruskal Wallis tests were employed (*P*<0.05).

**Results:**

The buccal position of the IMbCs was the most predominant (65.1%), and the location below the apical middle third of the lower incisor root was the most frequent (32.6%). No statistical significance was found between root resorption and location, level and impaction sector. Only mild root resorption and impaction sector 5 was significant (66.70%; *P*<0.001). The proximity of the follicle and crown of the IMbC did not affect the root structure of the lower incisors. 72.1% and 52.2% of mandibular canines did not present contact of the impacted canine or its follicle with the adjacent tooth respectively. Root lengths were similar in the different types of impactions (*P*>0.05).

**Conclusions:**

IMbCs are mostly positioned buccally and below the apical middle third of the root of the lower incisors. Likewise, the proximity of their follicles and crowns does not seem to affect the root structure of the lower incisors, producing minimal and infrequent root resorption.

** Key words:**Mandibular canine impaction, incisor root morphometry, cone-beam CT.

## Introduction

Tooth impaction is a condition in which there is an obstacle to the eruptive development of a tooth (1,2). The possibility of impaction can occur with any type of tooth in the dental arch; however, the teeth with the highest incidence of impaction, after the third molars, are the canines (2). The prevalence of maxillary canine impaction has been reported to be 0.2% to 5% and 0.31% to 1.35% for mandibular canines (3-5), although the incidence for mandibular canines has increased in in recent years (3,4,6,7).

Impacted mandibular canine (IMbC)) is a rare eruptive disorder (6). Sometimes the IMbC migrates across the midline in a process known as transmigration (8,9), with a prevalence ranging from 0.33% to 0.46% and with a varying severity (4,6,7,9-11).

Several factors such as space deficit, absence of lateral incisors, incisor root relationship, supernumerary teeth, premature loss of the primary dentition, retention of the deciduous canine, excessive crown size of the permanent canine, hereditary factors, functional distortions of the endocrine glands, tumors, cysts and trauma have been described as being related to the etiology of IMbC (9,12-14). In the mandibular bone, IMbC can persist for several years without causing symptoms (15).

Treatment of IMbC includes periodic check-ups, extractions and space closure, as well as surgical exposure and orthodontic traction, including autotransplantation (2,16). However, although it depends on the comprehensive diagnosis of the malocclusion, the position of the canine and its relationship with the neighboring teeth has a great influence on treatment decision making (17). In this perspective, a two-dimensional clinical radiographic diagnosis is not sufficient to evaluate the exact proximity of the IMbC to the apices of the mandibular incisors in the three planes of space. Therefore, cone beam computed tomography (CBCT) is required for surgical and biomechanical decision making when the treatment of choice is orthodontic traction (17,18).

The role of the mandibular canines in the dental arch is very important. They not only determine the structural conformation of the arch maintaining its length, but they are also of vital importance for a balanced and dynamic function of the occlusion, which together with their antagonist provide adequate conditions for the movements of laterality and canine disocclusion. The function of the mandibular canines in stomatological functional dynamism is directly proportional to their context and anatomical development, which cannot ideally be substituted by another dental piece. Therefore, repositioning of these teeth in the dental arch is fundamental, provided that the viability of their repositioning and the stability of the adjacent dental and anatomical structures are preserved. For functional, periodontal and esthetic purposes treatment of IMbC is justified, making it important for therapeutic purposes to determine the relationship of the IMbC with the apices of the incisors, since the prognosis and viability of orthosurgical traction may depend on this relationship.

Some studies have linked the location of the IMbC to the roots of the incisors (18-20). Many non-transmigrated impacted canines present a relationship with the middle third root of the adjacent teeth, and the transmigrated teeth have a relationship below the cervical middle third, indicating a predisposing factor to transmigration (21). There is little three-dimensional (3D) evidence associating the relationship of the mandibular incisors with lower canine impaction and this information is necessary for orthodontists to determine the most adequate treatment for these complex and increasingly more frequent cases. Therefore, the purpose of this study was to determine the association between the position of the IMbC with the morphometry of the roots of the lower incisors using a 3D technique.

## Material and Methods

This descriptive and retrospective study was approved by the Ethics Committee of the School of Dentistry of the Universidad Científica del Sur Lima, Peru (registration number 247-2021-POS8). The sample for this study was collected from private radiology centers in Peru, Colombia and the Dominican Republic. The CBCT studies evaluated were indicated by orthodontists and surgeons for diagnostic reasons unrelated to this research.

The CBCT images were taken between January 2018 and May 2021. The inclusion criteria were CBCTs of the antero-inferior sector presenting impacted mandibular canines related to the lower incisors of patients older than 11 years of age. Exclusion criteria included CBCTs associated with odontogenic and non-odontogenic lesions, patients with systemic diseases or associated craniofacial syndromes, with absent lower incisor adjacent to the impacted canine, and impacted canines in relation to posterior teeth.

Sample calculation was performed using a formula to estimate a proportion, considering an approximate population of 100 IMbCs, with a confidence level of 95%, a precision of 5% and a proportion of impacted canines in direct relation to the apex of their adjacent teeth that generated root resorption of 5% taken from a previous pilot study and similar to a published study (12). Although the required sample was 42 IMbCs, 43 IMbCs were collected.

Next, Digital Imaging and Communications in Medicine (DICOM) files were requested from computed tomography scanners with similar characteristics and a field of view of at least 5 x 5 or larger to measure heights, widths and depths. The DICOM files of the specimens collected were transferred for multiplanar reformatting using Dolphin Imaging and Management Solutions software, Patterson Dental Supply, Inc. Chatsworth, CA. Version 11.9.

**Figure 1:**
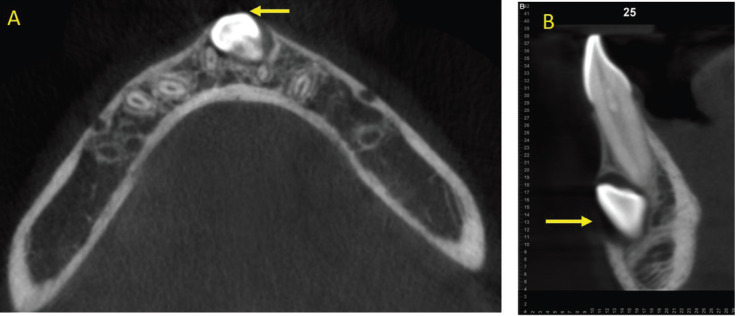
A,B) Identification of the location of the impacted canine in axial and sagittal planes, taking the alveolar cortices as a reference.

**Figure 2:**
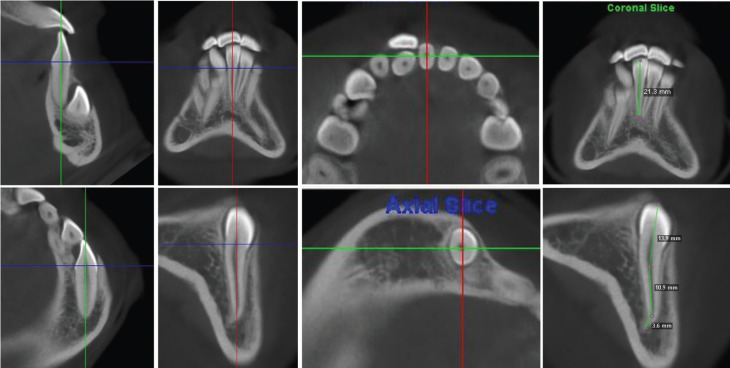
Longitudinal references of the lower incisors and impacted mandibular canine, in sagittal, frontal and axial planes, to measure the length from the incisal edge to the dental apex.

For reference taking and standardization of the sample, the impaction characteristics of the mandibular canines, sector and level of impaction, alpha angle (a), beta angle (b) and distance to the occlusal plane (d) were determined using the parameters previously proposed by Ericson and Kurol, adapted to similar anatomical points in the mandible (21). The dental midline and the central point of the chin were taken as references as the central reference of the mandible and the impacted sectors were delimited by taking the mesial and distal limits of the lower incisors and their longitudinal axes. Using axial and sagittal slices of the mandibular anterior area, the canines were classified according to their impaction location in buccal, bicortical and lingual sites in relation to the bony cortices. Likewise, measurements from the incisal edge to the root apex were obtained in millimeters of the dental length of mandibular incisors and impacted mandibular canine (Figs. 1,2).

**Figure 3:**
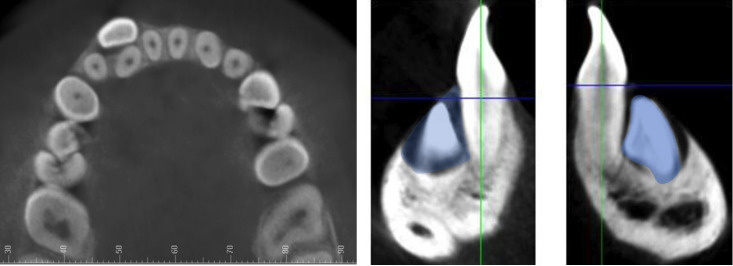
Relationship of the pericoronal sac/crown of the impacted mandibular canine with the root of the adjacent incisor, in axial and sagittal slices.

Axial, coronal and sagittal tomographic sections were used to examine the presence or absence of bone resorption in mandibular incisors directly related to the impacted canine, and the integrity of the periodontal ligament space or the interruption of its anatomical continuity. No resorption was considered when the periapical morphology was unaltered; Slight, a little resorbed periapical morphology; moderate: root resorption without pulp chamber invasion; severe: root resorption with pulp chamber invasion. We also assessed the relationship of the pericoronal sac and the crown of the impacted mandibular canine with the adjacent incisors, reporting contact at a distance of 0 to 0.5 mm, and no contact when greater than 0.5 mm (Fig. 3).

The evaluation of the data was performed by a specialist in oral and maxillofacial radiology with more than 15 years of experience, and a master’s degree student in oral and maxillofacial radiology, who was trained by the former. In addition, the calibration was performed by means of the intraclass correlation coefficient test (ICC=0.83) and Dalhberg’s formula (D= 0.64) to measure the reproducibility and agreement between intraoperator measurements in continuous data and the Kappa index (K= 0.80) to refer the agreement in categorical data.

-Statistical analysis

Statistical analysis was performed using SPSS version 26 statistical software (IBM Corp., NY, USA). The data were reflected in percentages and frequencies for categorical variables and in means with their respective standard deviations for continuous variables. Subsequently, associations between the type, sector, level and proximity of impaction with the condition of root resorption were evaluated with the Chi-square test. Normality of continuous data was assessed with the Shapiro-Wilk test, and root lengths were then compared between impaction types, sectors and levels with the Kruskal Wallis test. Differences were considered statistically significant when the *P* value was < 0.05.

## Results

This study evaluated 35 individuals, 17(48.6%) of whom were female and 18(51.4%) were male, with a mean age of 14.37± 10.26 years. Eight (23.5%) of the cases were bilateral and 27 (79.4%) were unilateral, for a total of 43 impacted mandibular canines (22 left and 21 right canines). The type of impaction was evaluated according to location, the most frequent being buccal (28; 65.1%), followed by bicortical (8;18.6%) and lingual (7; 16.3%). According to their relationship with the midline, the sectors most frequently impacted were sector 1 (20.9%), sector 3 (18.6%) and sector 7 (18.6%). In relation to the lower incisors in the vertical direction, level 1 (coronal) was most frequent with 32.6%, followed by level 3 (radicular) and level 4 (apical) both with 25.6% (Table 1).

**Table 1:**
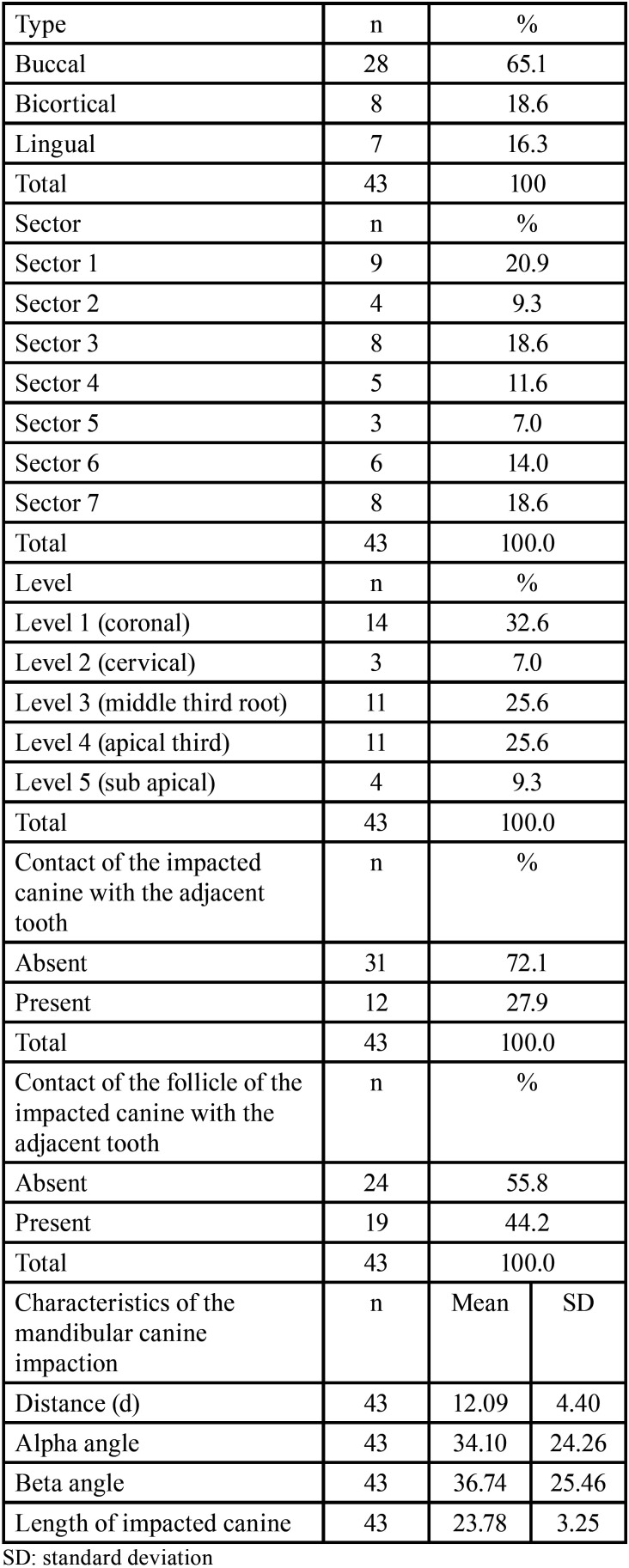
Location of impacted mandibular canines in the sample evaluated.

The relationship of the impacted canines with the lower incisors showed that the absence of contact of the crown or the follicle of the IMbC between the two teeth was more frequent (72.1%), with slight contact being observed in the remaining percentage (27.9%). The impaction severity data showed that the mean distance of the IMbC cusp from the occlusal plane was 12.9 + 4.40 mm; alpha and beta angle adaptation for the mandible was 34.10 + 24.26 and 36.74 + 25.46 degrees, respectively, and the mean IMbC length was 23.78 + 3.25 mm (Table 1).

No statistical significance was found between root resorption of the lower incisors related to location, level and impaction sector (Table 2- Table 4). Only mild root resorption (slight loss of contour of the periodontal ligament) and impaction sector 5, which was associated with inclination and displacement in contact with the midline, showed statistical significance (66.70%; *P* < 0.001) (Table 3). The length of the mandibular incisors and IMbC showed no relationship with the degree or severity of impaction (*P* >0.05) (Table 5- Table 7).

**Table 2:**
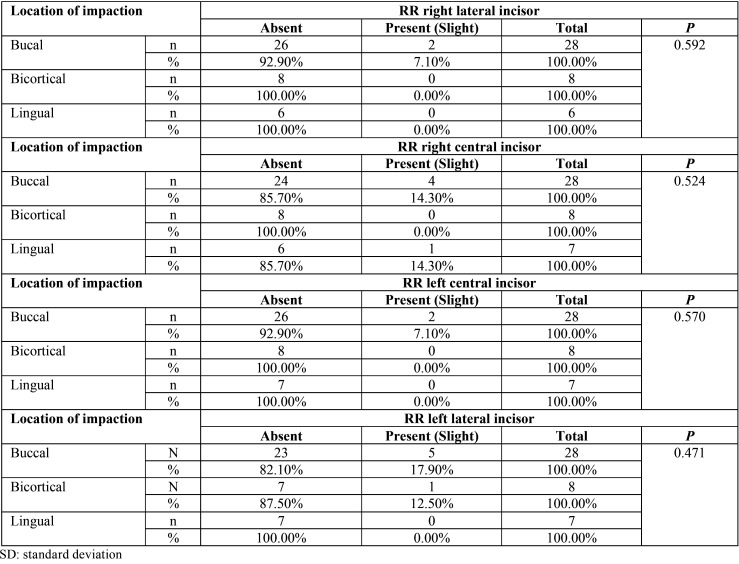
Association between location of impaction of IMbCs and the condition of root resorption (RR) of the lower incisors.

**Table 3:**
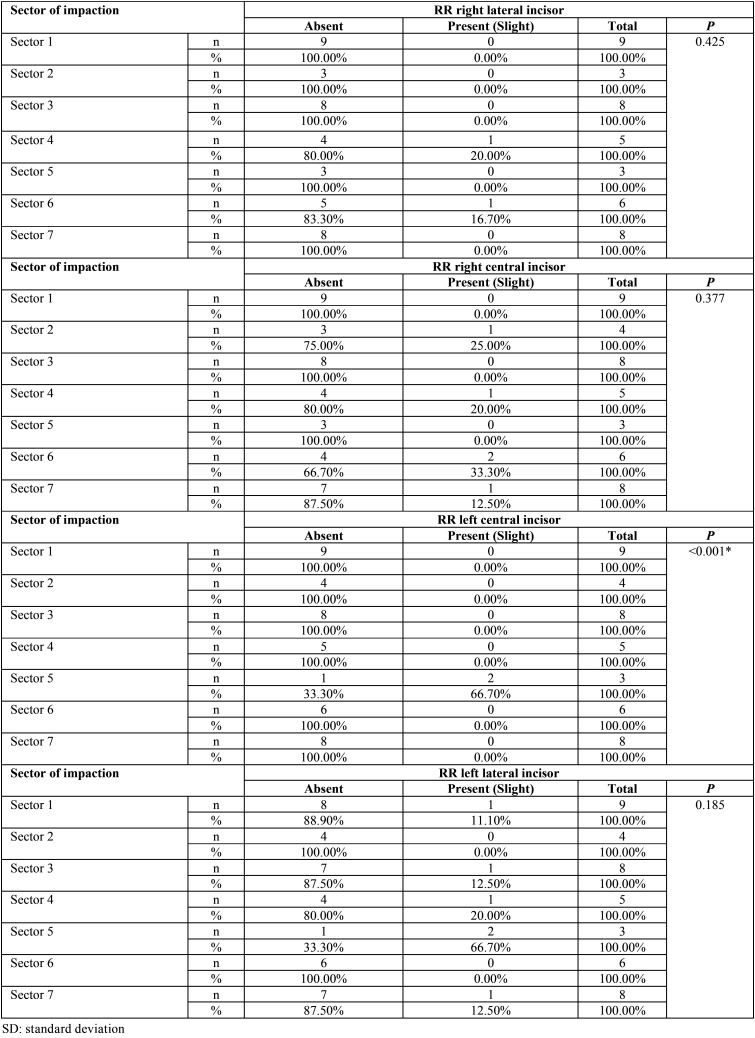
Association between the impaction sector of the mandibular canines and the condition of root resorption (RR) of the lower incisors.

**Table 4:**
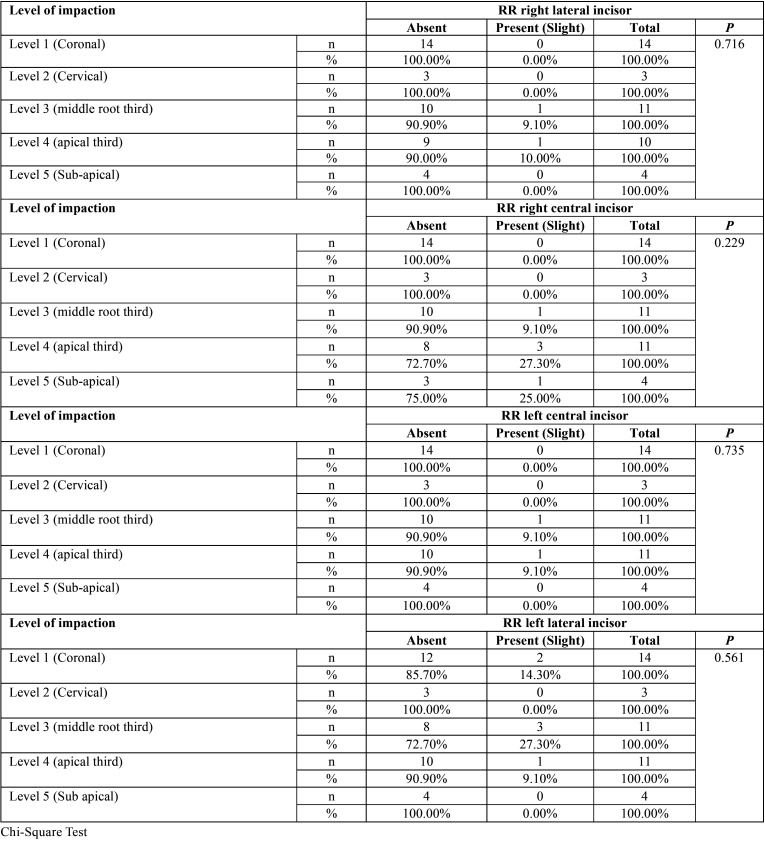
Association between the level of mandibular canine impaction and the condition of root resorption (RR) of the lower incisors.

**Table 5:**
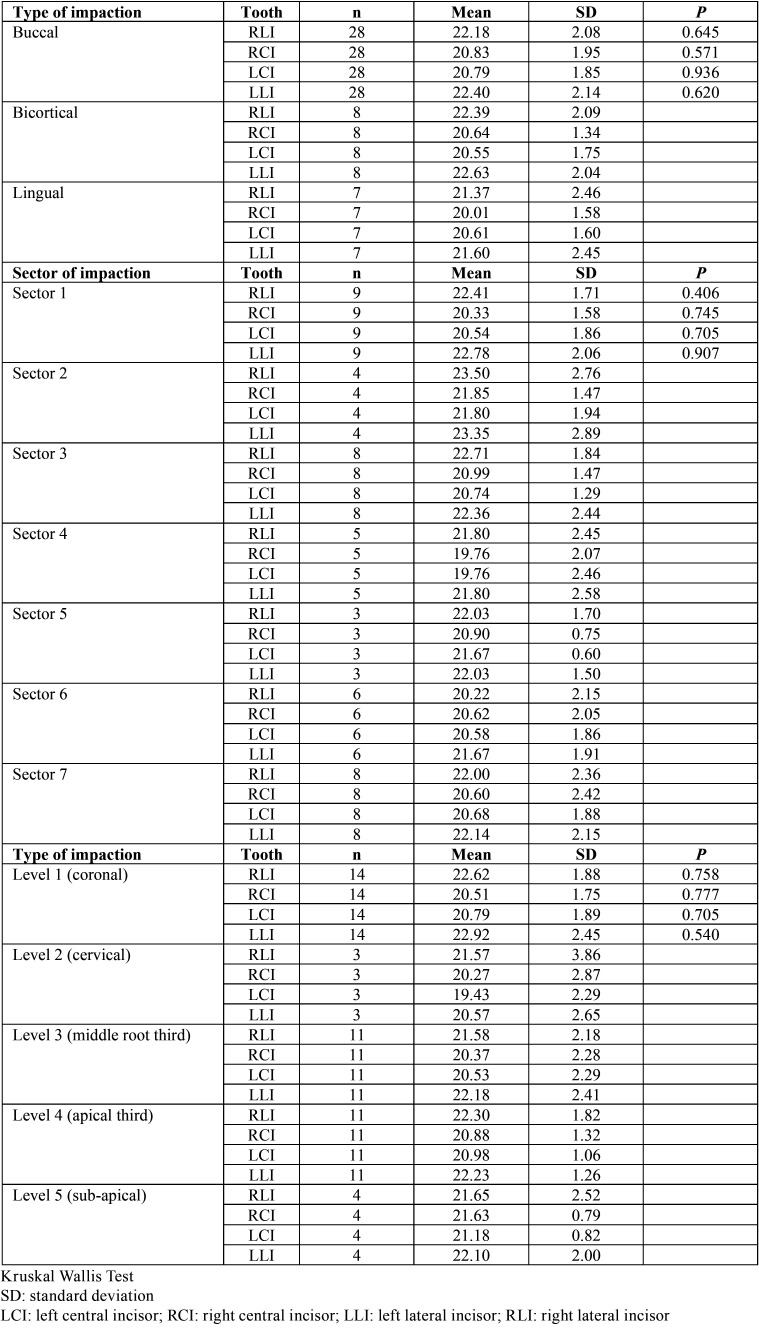
Comparison of the root length of the lower incisors according to the type, sector and level of impaction of the mandibular canines.

**Table 6:**
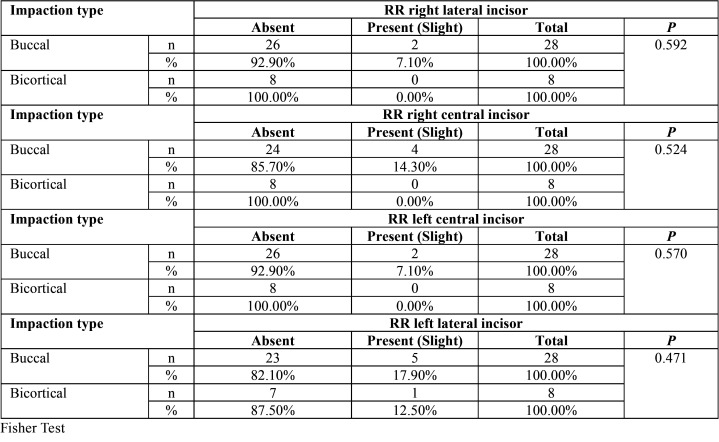
Association between impacted mandibular canine crown contact and the condition of root resorption (RR) of the lower incisors.

**Table 7:**
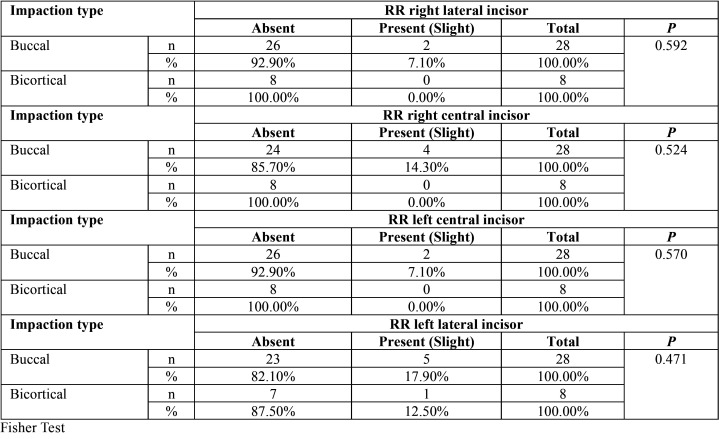
Association between impacted mandibular canine follicle contact and the condition root resorption (RR) of the lower incisors.

## Discussion

The influence of impacted canines on the surrounding tissues and migratory changes may differ from jaw to jaw. Many studies have focused on maxillary canines and very few on IMbC. Although the incidence of mandibular canine impaction is low (7,22), the evaluation of the impaction characteristics is very important for clinical diagnosis and determination of an appropriate treatment plan. IMbCs usually behave differently in aspects such as migration, location, malposition and alteration of neighboring tissues when compared to impacted canines in the maxilla. The incidence of impacted canines in the mandibular bone, as well as their bone density, the absence of sutures and pneumatic cavities suggest a different behavior. Ectopic migration in the contralateral direction crossing the midline through the subapical basal bone of the chin is typical of IMbC as is direct contact of the pericoronary follicle with the apices of the incisors. In this sense, the objective of this study was to three-dimensionally determine the relationship between the position of the IMbC with the position and morphometry of the lower incisors.

IMbC has been reported to occur more frequently in women than in men, and more commonly unilaterally, with the left side being more involved (4,23,24). However, in our study, neither sex, nor the left or right quadrant influenced the results.

With the use of 3D images, the location of the crown of the impacted canine can be determined in three directions: linguo-labial, vertical and disto mesial. The linguo-labial position facilitates precision for the surgical or orthodontic approach. According to Bertl (12) and Sajnani (25), labial impaction of the IMbC is 2-fold more frequent than lingual impaction. Furthermore, anatomical and histomorphometric factors of the mandibular bone epidemiologically influence the buccal, intermediate and lingual location of IMbC when compared to impacted maxillary canines. In our study 65.1% were buccal, 18.6% intermediate and 16.3% lingual. According to Ericson and Kurol (26,27), impacted maxillary canines are located more palatally (85%) than buccally (15%). In the present study, the most frequent location of impacted mandibular canines was evidently buccal, in contrast to the findings of Cakir Karabas *et al*. (5), who reported the bicortical location as the most frequent.

In the vertical position, the height of the canine cusp was frequently presented at the coronal level (32.6 %) of the incisor root, followed by the mid-root (25.6 %) and apical level (25.6 %). Together these last two may coincide with the middle and apical frequency reported in the literature (5,12,23), contributing to possibly avoiding a higher prevalence of IMbC transmigration.

The distance of the canine from the midline determines the impacted sector or its disto mesial position in the anterior sector. For the purpose of seeking the standardization reported in the literature, the classification of the severity of canine impaction by Kurol and Ericson (28) for maxillary canines was used. However, this classification should be applied with caution in mandibular canines due to the structural and anatomical variations of the mandibular bone in which there are no sutures, no middle raphe, no pneumatic cavities, the chin is solid and the bone density of the dentoalveolar and basal bone is very different from that of the upper jaw. In this study the most frequent sectors were sector 1 or the ideal position in 20.9% of the cases, sector 3 at the level of the lateral and central incisor in 18.6% and sector 7 crossing the midline in 18.6%. These results had no impact on the generation of apical resorption.

Migration of the canine is commonly but is considered transmigration when passing the midline, having a prevalence of 0.46% (7). The literature includes cases specifically in mandibular canines because it is a characteristic phenomenon of their impaction. The criteria for mandibular midline references are another controversial factor, since it is necessary to perform radiographic repairs of the chin, such as the lingual foramen, the chin protuberance, the geni apophyses, the midpoint of the interincisive bony crest or some sign of what was once the symphysis of the chin as described in previous studies (12). In the present study the interincisive dental midline was taken as a reference together with the radiographic anatomical landmarks described above. It has been reported that transmigrated mandibular canines are located more subapical to the incisors compared to non-transmigrated canines that are related to the incisor roots. Transmigration is also associated with greater horizontality of the IMbC (4,25). In the present study, evaluation of the inclination of the IMbC with respect to the incisors showed that the inclination did not exceed 60 degrees and the horizontality was not less than 10 degrees, thereby reducing the risk of transmigration.

Contact of the canine apex with the mandibular basal produces a decrease in migration toward the midline. The possibility of transmigration of mandibular canines may be related to the absence of a suture in the mandibular midline, which allows free movement of the tooth germ through the alveolar bone. Many transmigrations occur during root development of the canine, when the length and apex are not finalized. These teeth are characterized by having greater viability for mesial migration, which is why radiographic evaluation of the dentition is recommended between 8 and 9 years of age. In addition, cases of transmigration have been reported as early as 8 years of age, and it is debated whether the older the age, the greater the predisposition to transmigration (4,17,26). In this study, the average age of impacted canines in sector 7 with transmigration features was 11.5 years with a closed apical foramen. Another important characteristic derived from the results of the present study was the absence of association between tooth size and the severity of mandibular canine impaction, which allows ruling out the suggestion that the length of adjacent incisors influences IMbC migration toward the midline. Nevertheless, future morphological studies in this regard are necessary to clarify this finding.

The relationship of the pericoronary follicle of impacted maxillary canines to root resorption of upper incisors has been described by 3D imaging (29). Several studies have attempted to explain the chemical proximity of the pericoronary dental follicle of normal erupting to deciduous teeth, as well as that of impacted ectopic canines to neighboring incisors, but the difference in the resorptive process between maxillary canines and mandibular canines has not yet been clarified (30,31). In the literature it has been described that the width of the maxillary canine follicle ranges from 0.0mm to 14.1mm + 2.4 mm (27). The variation in the dimensions of the pericoronary sac depends on several factors, such as the quality of the alveolar bone, the space available for the development of the dental follicle and the changes produced by hormones or any factor related to growth and development (27,32). It has been reported that there is no statistically significant relationship between the width and shape of the tooth follicle and the transmigration of IMbC12 and root resorption of teeth adjacent to impacted maxillary canines (33-36). Many of these studies have shown that even when the tooth follicle is in contact with the adjacent incisor root, eruption of the maxillary incisor occurs without adverse effects (37,38). However, Chaushu *et al*. (39) reported that impacted maxillary canine follicles wider than 2 mm may be a risk factor for the resorption of adjacent teeth. Although the width and shape of the follicle of the impacted maxillary canine did not appear to have statistical significance with respect to root resorption of adjacent teeth, Ericson and Bjerklin (38) reported that the periodontal contour can be slightly affected in most cases without producing root resorption. In our study, contact of the tooth follicle and the IMbC crown enamel was found in several of the canines in the sample. However, the degree of root resorption was minimal in the periodontal ligament space and very tangentially contacted the root cementum. Nineteen impacted canines were found in slight contact of the follicle with an adjacent tooth. In the present study, the most frequent IMbC contact was with the left lateral incisor, being more severe in two of the cases close to the midline in the labial position. Bertl *et al*. (12) reported that the cases of resorption of their specimens occurred in lingually impacted canines, with no relation to follicle size or shape. Cakir Karabas *et al*. (5) reported that most of the canines in their sample did not cause resorption. Ericson and Bjerklin (38) found that the incisor roots that presented root resorption were observed. when the impacted canine crown was in contact with the incisor root. Despite all these findings, the incidence of root resorption due to impaction, which is frequent in maxillary incisors, does not occur in the same way in mandibular incisors.

Mazinis *et al*. (17) found that there was no significant association between transmigrated canines and resorption of adjacent teeth. However, they highlighted that most periodontal discontinuities were more common in non-transmigrated impacted canines, suggesting that contralateral migration of canines does not cause adjacent teeth to be affected, similar to what was described in the Koc study (19).

3D analysis of canine impaction is important for the evaluation of impaction characteristics, as it provides greater certainty of diagnosis of the impacted tooth. Studies with large sample sizes are needed to evaluate the relationship of follicle width and shape, surrounding bone, adjacent anatomical structures, hormonal influence, growth factors and genetics in relation to impacted canines of the lower jaw, as more evidence is needed to support the frequency of transmigrations and there is almost null incidence of root resorption of incisors due to IMbC. Given the low prevalence of IMbC, it was necessary to select multicenter samples from different tomographs, but with a similar field of view. The use of DICOM files allowed 3D digital exchange of multicenter CBCTs without substantially affecting the accuracy of the measurements within a single software (Dolphin Imaging). The sample size was also affected because the IMbCs selected had to have some specific relationship of the follicle and/or crown to the roots of the lower incisors. The preventive therapeutic approach to mandibular impaction and transmigration should be considered more frequently; likewise, orthodontic- and surgically-guided forced eruption should be analyzed with the purpose of making IMbC more viable in the buccal mesoangulated position and with a low risk of root resorption.

## Conclusions

IMbCs are mainly positioned buccally and below the apical middle third of the root of the lower incisors. Likewise, the proximity of their follicles and crowns do not seem to affect the root structure of the lower incisors, producing minimal and infrequent root resorption, all of which should be taken into account in clinical practice.
